# HIV-1 Protease: Structural Perspectives on Drug Resistance

**DOI:** 10.3390/v1031110

**Published:** 2009-12-03

**Authors:** Irene T. Weber, Johnson Agniswamy

**Affiliations:** Department of Biology, Molecular Basis of Disease Program, Georgia State University, Atlanta, GA 30303, USA; E-Mail: jagniswamy@gsu.edu

**Keywords:** protease inhibitors, drug resistance, aspartic protease, molecular mechanism, darunavir

## Abstract

Antiviral inhibitors of HIV-1 protease are a notable success of structure-based drug design and have dramatically improved AIDS therapy. Analysis of the structures and activities of drug resistant protease variants has revealed novel molecular mechanisms of drug resistance and guided the design of tight-binding inhibitors for resistant variants. The plethora of structures reveals distinct molecular mechanisms associated with resistance: mutations that alter the protease interactions with inhibitors or substrates; mutations that alter dimer stability; and distal mutations that transmit changes to the active site. These insights will inform the continuing design of novel antiviral inhibitors targeting resistant strains of HIV.

## 1. Introduction

The structures and activities of HIV protease and its drug-resistant variants and their interactions with inhibitors have been studied for nearly 20 years in order to combat the challenges of AIDS antiviral therapy and the evolution of HIV drug resistance [1]. About 25 different antiretroviral drugs (ARV) are currently used in the combat against human immunodeficiency virus (HIV) and acquired immunodeficiency syndrome (AIDS). ARVs are divided into five different classes depending on their viral target. Currently recommended AIDS therapy employs a mixture of drugs from different classes in highly active antiretroviral therapy (HAART). Nine of these drugs target HIV protease and belong to the class of protease inhibitors (PIs). The first PI was introduced into clinical practice in 1995 when therapy became possible with drugs blocking the two viral enzymes, protease and reverse transcriptase. The inclusion of PIs in antiviral therapy has resulted in major clinical benefits including prolonged viral suppression, control, reduced morbidity and mortality for HIV infected people [2–4]. Encouraged by the potency and efficacy of the antiviral PIs, more efforts are underway to come up with the next generation of inhibitors [5,6].

However, the emergence of drug resistance to PIs has compromised the effectiveness of treatment of HIV infections [7–9]. Newer HIV infections that transmit drug resistant virus into drug naïve patients limit antiviral options and thus complicate the management of HIV infection [10–12]. More mutations are selected by PIs than any other class of ARV, partly due to their use for over a decade. The drug resistance to a particular PI also leads to cross resistance to other drugs within the PI class. The degree of cross resistance depends on the number of mutations and the type of mutation selected by a PI [13]. With the availability of 9 PI drugs it is still possible to salvage a response from a different PI following failure of treatment with the first PI. Ritonavir is now solely used as a pharmokinetic booster and ritonavir boosted lopinavir/r, atazanavir/r, fosamprenavir/r and saquinavir/r are used as first line therapy against HIV infection [14,15]. Unboosted nelfinavir, atazanavir and fosamprenavir are suboptimal alternative choices for first line therapy. Boosted lopinavir/r, tipranavir/r and darunavir/r are used for salvage therapy for drug resistant mutants. Though ritonavir boosted PIs have limited the development of PI drug resistance, the presence of particular mutations indicate that a particular PI may not be effective [15]. Due to the challenging problem of drug resistance it is especially important to understand the mechanism of PI drug resistance at the molecular level. This knowledge can be employed to design the next generation of PIs with improved potency and efficacy, not only against the wild type HIV, but also against drug resistant strains. In this perspective, we describe current knowledge of the molecular mechanisms of PI drug resistance deduced from the available crystal structures of wild type HIV protease and its drug resistant mutants in their complexes with PIs.

## 2. HIV protease and structure guided design of PIs

HIV protease plays a critical role in viral maturation for producing infectious virus particles. The protease cleaves the precursor Gag and Gag-Pol polyproteins at a minimum of 9 distinct sites. The cleavages release the structural proteins matrix, capsid, and nucleocapsid, spacer peptides p1, p2, and p6, and functional enzymes reverse transcriptase, protease and integrase. Alteration of protease activity leads to defective viral particles and reduced infectivity [16,17]. Inactivation of HIV protease resulting in the lack of infectious virus has made protease an attractive drug target for HIV and AIDS [18,19].

Structure-guided inhibitor design has proved to be a powerful technique for discovering drug candidates with successful development of HIV protease inhibitors for AIDS therapy [20], protein kinase inhibitors for cancer therapy [21], and neuraminidase inhibitors for treatment of influenza virus infection [22]. Proteases are valuable targets for structure-based drug designs with therapeutic success for inhibiting aspartic proteases like renin as well as HIV protease [23]. Other examples of protease inhibitors in clinical trials include memapsin inhibitors in Phase II trials for Alzheimer’s Disease [24].

**Figure 1.**
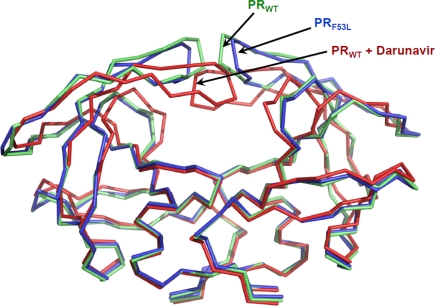
Structures of HIV-1 protease dimer. Superposition of unliganded protease (PR_WT_ in blue, PDB ID: 1HHP [29]), unliganded protease with F53L mutation (PR_F53L_ in green, PDB ID: 2G69, [28]) and protease complex with darunavir (red, PDB ID: 2IEN, [30] inhibitor is removed for clarity). The unliganded structures exhibit opened flap conformation, while the protease flaps form the closed conformation with darunavir.

Knowledge of the crystal structure of HIV-1 protease and its recognition of substrate analog inhibitors was critical for the design of antiviral PIs. The HIV protease is an aspartic protease and the catalytic site has the characteristic Asp-Thr-Gly sequence common to all aspartic proteases. It functions as a symmetric homodimer consisting of 99 amino acids per monomer. The structure of protease is predominantly β-sheet with the two aspartic acids Asp-25 from the two monomers forming the central active site. The three important regions in the protease structure are the active site cavity, the flexible flaps, and the dimer interface. PIs are competitive inhibitors that bind at the active site of the protease with the flaps folded into a closed conformation over the active site. The dynamics of the flap region is important for the activity of the enzyme. The flaps were observed in closed and open conformation in the crystal structures of inhibitor bound and free protease (Figure 1) [25–29]. This opening and closing of the flaps enables the substrate to enter and leave the active site of protease. Inhibitor bound at the active site with the flaps in closed conformations keeps the enzyme in a locked down state and prevents the processing of substrates.

**Figure 2.**
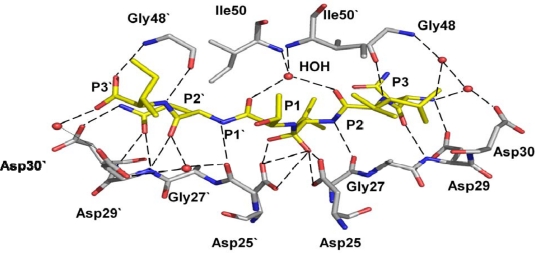
Hydrogen bond interactions of the peptide tetrahedral intermediate with HIV-1 protease (PDB ID: 3B7V [32]). The peptide intermediate is in yellow bonds and the protease in grey. Hydrogen bonds are indicated by broken lines. Interactions with the catalytic Asp25 and 25’ are omitted for clarity.

The initial structural information for design of antiviral inhibitors was derived from complexes of wild type HIV-1 protease with non-hydrolysable peptide analogs. These peptide analogs share the sequences of natural cleavage sites in the Gag and Gag-Pol polyproteins, however, the hydrolysable bond is replaced by the reduced peptide group (CH_2_-NH) or hydroxyethylamine (CHOH-NH). Common features for protease-peptide interactions were deduced from these crystal structures [31]. HIV-1 protease recognizes peptides of at least six residues long from P3 to P3’ and catalyzes hydrolysis of the P1-P1’ peptide bond. The peptide extends inside the protease dimer and interacts with both subunits on either side of the two catalytic Asp25 residues in the central catalytic site. The peptide is bound between the active site residues 25–29 and two flaps by means of conserved hydrogen bonds connecting the main chain amide and carbonyl oxygens of the peptide, active site and flaps. The carboxylate side chain of Asp29 also forms hydrogen bonds with the peptide amide. The peptide side chains (P4-P3’) lie in consecutive pockets or subsites (S4-S3’) formed by protease residues. Recently, crystal structures have revealed the tetrahedral reaction intermediate of a peptide trapped in the active site of HIV-1 protease [32,33]. These structures demonstrate similar hydrogen bond interactions of the protease with reaction intermediates as described for peptide analog inhibitors. The protease hydrogen bond interactions with a tetrahedral intermediate peptide are shown in Figure 2 [32]. The protease active site cavity comprises residues Arg8, Leu23, Asp25, Gly27, Ala28, Asp29, Asp30, Val32, Lys45, Ile47, Met46, Gly48, Gly49, Ile50, Phe53, Leu76, Thr80, Pro81, Val82, Ile84. The majority of the residues forming the substrate binding site are hydrophobic; the exceptions are the catalytic Asp25 and Asp29, which form hydrogen bonds with peptide main chain groups, and Arg8, Asp30 and Lys45 which can interact with polar side chains or distal main chain groups in longer peptides [34].

The substrate specificity of HIV-1 protease has been studied extensively, as reviewed [35]. Knowledge of substrate recognition has benefited greatly from comparative analysis of other retroviral proteases, as reviewed in an accompanying article in this book [36]. Information on protease recognition of substrates has been incorporated into the design of antiviral inhibitors, which contain polar groups that form hydrogen bonds with the protease main chain and large hydrophobic groups replacing the P1 and P1’ peptide side chains.

The clinical PIs incorporate the common features deduced from the crystal structures of HIV protease with peptidic inhibitors. Saquinavir was the first drug approved for HIV protease; indinavir and ritonavir were introduced soon afterward. The PIs were designed to bind tightly to the wild type enzyme by mimicking the transition state of substrates [37]. The binding affinity of the PIs varies from nanomolar to picomolar. In the current stage, as exemplified by the recently developed drugs tipranavir and darunavir, the strategy was to target drug resistant variants of the HIV-1 protease. The PIs are generally shorter than the peptide substrates and contain hydrophobic groups that bind within the hydrophobic pockets at the S2-S2’ subsites of protease. The early PIs were designed with polar groups resembling those of the substrate peptide main chain, and include a central hydroxyl that interacts with the catalytic aspartates and mimics the hydroxyl of a tetrahedral reaction intermediate. The later inhibitors amprenavir, tipranavir and darunavir were designed with less peptidic backbone features but retaining the central hydroxyl group [38,39]. Sulphonamide replaces the peptide like carbonyl in the earlier inhibitors of protease.

## 3. Evolution of Drug resistance to PIs

**Figure 3.**
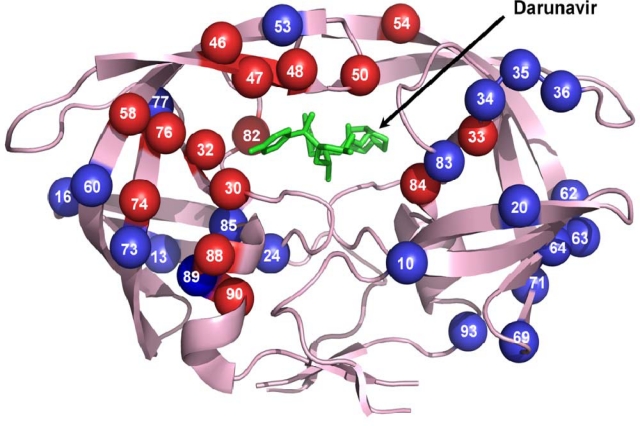
Sites of the resistance mutations on protease dimer. The protease dimer is in pink ribbons with darunavir in green sticks. Major and minor resistance mutations are colored as red and blue spheres, respectively. Mutations are distributed on both the monomers to increase visibility.

Introduction of the first PI, saquinavir, in 1995 marked an important advance in the treatment of HIV infection. Then, it was possible for more effective therapy using a cocktail of drugs to target the protease as well as the reverse transcriptase. However, resistance to the PI quickly emerged [40]. These resistant mutations reduced susceptibility to the PI while maintaining protease function [13]. Resistance is seen for all the drugs currently used in HAART, however, PIs appear to select more mutations than other classes of drugs. Drug resistant mutations of the protease have emerged against all the clinically available PIs (Table 1). Though HAART is successful in suppressing the viral replication it cannot completely eliminate the integrated viral DNA. Thus long term use of drugs is essential. Adherence to therapy greatly influences emergence of drug resistance [41–43]. Drug side effects and toxicity are major reasons for loss of patient compliance and viral failure [44,45]. In addition, rapid mutation due to high rate of viral replication and lack of nucleoside proof reading in HIV assists emergence of drug resistance. Also, the number of primary infections involving transmission of PI drug resistant strains is on the rise further hindering the treatment process [46]. Although PIs select for specific mutations, the resistant mutation pattern for each PI is complex and difficult to predict [47,48]. Almost 50 different residues are mutated even in the absence of an inhibitor forming a large background of neutral mutations in the relatively small protease [49]. According to the International Aids Society-US panel for ARV resistance, mutations in 37 of 99 residues in HIV protease have clinical relevance to drug resistance among the current PIs (Figure 3) [50]. These mutations are classified into major and minor mutations depending on their effect in antiviral therapy. Seventeen mutation sites are considered major mutations that render high levels of drug resistance to one or more PIs. Major resistance mutations are generally selected early and are much more inhibitor specific. Most of the minor mutations are considered to act as accessory mutations and compensate for the replication impairment due to the major drug resistance mutations. Furthermore, resistance mutations in the protease can be accompanied by mutations in the viral polyprotein cleavage sites.

Mutations in the Gag precursor cleavage sites, NC/p1 and p1/p6, are strongly associated with resistance to protease inhibitors [14]. Also, protease drug resistance can emerge due to mutations in the Gag substrate alone, rather than in the enzyme [51]. In addition, recent reports indicate a role for amino acid insertions in the drug resistance of HIV protease [52–54].

Drug resistance is acquired by accumulation of a number of resistance mutations, although the presence of one or two major mutations significantly increases resistance to a PI. Different PIs select for different major mutations, although there is much overlap leading to cross resistance. Some mutations that result in loss of PI susceptibility alter residues at the active site cavity and flap regions (Table 1). Among the 15 sites of major resistance mutations 8 alter residues forming the active site cavity. However, mutations at distal regions (not in the active site cavity or flap) are also selected as major mutations by PIs. In the case of lopinavir, high levels of resistance are associated with specific mutations at positions 32, 47 and 82 in the active site cavity [55]. The distal mutation of L90M appears as a major resistance mutation for saquinavir and nelfinavir, while another distal mutation N88S is selected by atazanavir. Some of the major resistance mutations are more commonly associated with specific PIs, like D30N for nelfinavir, G48V for saquinavir, and V32I for lopinavir, while others can substantially affect the binding affinity of more than one PI [56]. I84V is a major resistance mutation for 5 of the PIs and has significant resistance to all the clinical PIs. Substitutions at residue 50 have major effects on susceptibility to atazanavir, darunavir and amprenavir. In addition, mutations at Val82 are selected by all the PIs, except darunavir. Despite these examples of cross resistance, no overall simple pattern appears to relate the mutations selected in resistance with the chemical structures of different inhibitors.

## 4. Molecular mechanisms of drug resistance

Studies of the structure and activities of HIV-1 protease variants reveal several molecular mechanisms associated with resistance arising from specific mutations. Resistance mechanisms may depend on the type of mutation and/or the drug used in therapy. The use of several PIs in HAART, however, complicates the analysis of drug-specific resistance mechanisms. A number of crystal structures have been solved of drug resistant protease variants in complex with the corresponding PIs, especially darunavir, indinavir and saquinavir. Table 1 indicates (in red) resistant mutations for which protease crystal structures are available. The analyzed protease variants include single and multiple mutations appearing in resistance. The changes due to a specific mutation are best understood in the context of the wild type protease sequence with a single substitution, rather than in a multiply mutated variant. Many structural studies have focused on darunavir due to its development for salvage therapy of drug resistant HIV. Detailed structural changes were discovered in high resolution and atomic resolution crystal structures, which have benefited from using synchrotron X-ray beamlines. In fact, the highest resolution to date is the sub-atomic resolution (0.84 Å) for the mutant of HIV protease (V32I) with darunavir [57]. The molecular mechanisms producing drug resistance are deduced from comparison of the structures and activities of the wild type and mutant proteases [58,1]. This accumulating knowledge has revealed several distinct mechanisms by which the virus evades the PI drugs. Generally, specific mutations show similar effects in complexes with several PIs. Moreover, the crystal structures of protease variants without inhibitor show some unique features contributing to drug resistance. Several categories have been identified in studies of protease with single substitutions: 1) active site mutations that alter direct interactions of the protease with inhibitor or substrate leading to reduced inhibition; 2) mutations at the dimer interface that alter protease stability; and 3) mutations of distal mutations showing a variety of effects. Some resistance variants fall into more than one category, or display other unusual changes in structure and/or activity. Examples are given in each category and the known Ki values for the inhibitors studied in these examples are listed in Table 2.

### 4.1. Reduced interactions with inhibitor

The simplest type of change results from mutation of a single residue in the active site cavity that eliminates direct interactions with inhibitor. Many of these resistance mutations are very conservative substitutions of hydrophobic amino acids, for example, substitutions among leucine, valine, isoleucine and methionine. The types of mutations that alter residues contributing to the inhibitor binding site have been widely studied. Residues Leu23, Asp30, Val32, Met46, Ile47, Gly48, Ile50, Val82, and Ile84 form interactions with substrates or inhibitors in the active site cavity and are mutated in drug resistance (Table 1).

**Figure 4.**
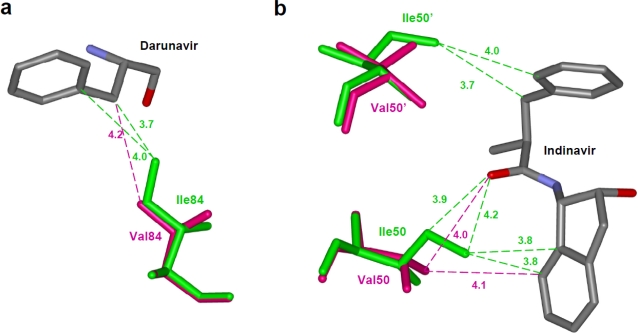
Resistance mutations showing loss of direct interactions with the inhibitor. (a) The I84V substitution results in loss of van der Waals contacts between residue 84 and darunavir. Wild type Ile84 (PDB ID: 2IEN) and mutant Val84 (PDB ID: 2IEO) are shown in green and magenta sticks, respectively. Only part of darunavir (grey bonds) is shown for clarity [30]. (b) Drug resistant mutation I50V is accompanied by loss of several interactions with indinavir [61]. Wild type Ile50 (PDB ID: 1SDT) and mutant Val (PDB ID: 2AVS) are shown as green and magenta sticks. Only the central portion of indinavir is shown in grey. The interatomic distances are given in Å.

One straightforward example of loss of interactions is seen for protease with the I84V mutation. In the crystal structure of the I84V variant in complex with darunavir, the mutation resulted in loss of two van der Waals contacts between the residue 84 and the inhibitor (Figure 4a) [30]. This loss of interactions with the inhibitor due to reduced size of the mutated residue resulted in an increased inhibition constant. The I84V substitution increases the K_i_ value for darunavir by 3-fold when compared with wild type protease. In contrast, the structure of I84V mutant protease with saquinavir displays little overall change in interactions with the inhibitor, consistent with the insignificant change in inhibition constant and designation of I84V as a minor mutation in saquinavir resistance [59].

Mutation D30N has a major effect in resistance to nelfinavir, although, it is uncommon in resistance to the other drugs. Unlike most tested mutations, the variant with D30N shows altered specificity for substrates representing different polyprotein cleavage sites [60]. Asp30 lies in the active site cavity and can form ionic or hydrogen bond interactions with peptide side chains or the ends of PIs like darunavir [30]. The D30N variant showed loss of a direct hydrogen bond with the aniline NH2 of darunavir and 30-fold reduced inhibition. However, D30N is not common in resistance to darunavir, probably due to defects in viral maturation resulting from its altered substrate specificity.

Structural studies of the I50V variant in complex with indinavir showed that the van der Waals interactions between the Ile CD and indinavir in the wild type are lost due in the mutant (Figure 4b) [61]. The wild type protease has 9 hydrophobic contacts between residues 50 from the two monomers and the inhibitor whereas the variant has lost four of those interactions. The I50V mutation also alters the dimer interface, as described for the next category of resistance mutations.

Flap mutation G48V, unlike most of the drug resistant mutations, increases the size of the amino acid side chain. G48V is a major mutation selected by saquinavir treatment and is cross resistant to atazanavir. Structural studies of G48V/L90M double mutant with saquinavir revealed that the number of interactions made by the flap region 47–50 with inhibitor is reduced relative to the wild type complex [62]. An important hydrogen bond between the carbonyl oxygen of residue 48 and the amide of saquinavir at P2 is also lost because of the increased side chain size of G48V.

These studies show that loss of direct protease interactions with inhibitor is a widely used mechanism producing resistance against PIs.

### 4.2. Main chain shift due to mutation

**Figure 5.**
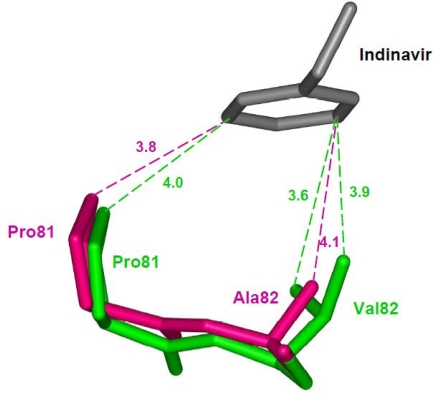
The V82A mutation shows a shift in the main chain atoms of residues 81 and 82 that partially compensates for the loss of interactions due to substitution of a smaller side chain [64]. The wild type Val82 (PDB ID: 1SDT) and Ala mutant (PDB ID: 1SDV) are shown as green and magenta sticks, respectively.

Val82 lies in the active site cavity and interacts with P1 and P1’ groups in peptides or inhibitors. Resistance mutation V82A is described separately since the structural effects include adaptation to maintain interactions with inhibitors. Structural studies of V82A in the context of the active wild type protease show similar small changes with indinavir, darunavir and saquinavir [30,63,34,59,64]. In contrast, in studies with inactive protease containing mutation D25N and V82A in complex with saquinavir, the protease has lost almost all the van der Waals interactions with the inhibitor [65]. In addition to hydrophobic contacts, some hydrogen bonds lost in this structure are probably due to the presence of inactivating mutation D25N in the double mutant structure. Mutant D25N alone shows significantly decreased dimer stability [66], which may permit drastic structural changes when combined with another mutation. In contrast, the crystal structure of mutant V82A in the catalytically active protease has more or less retained all the van der Waals contacts with indinavir [64]. A structural adaptation of residues 80–82 (the 80’s loop) accommodates the inhibitor. In the indinavir complex the main chain atoms of residues 81 and 82 are shifted to maintain similar interactions as for the wild type protease (Figure 5). Also, an identical mechanism of main chain shift due to V82A substitution is observed in the single mutation studies with saquinavir, darunavir and substrate analogs [59,30,34]. Unlike in darunavir and indinavir complexes, the main chain shift due to V82A in the saquinavir complex does not compensate for the loss of inhibitor interaction due to mutation [59]. The adoption of a similar structural mechanism in protease binding to saquinavir, indinavir and darunavir suggests why this mutation appears to be cross resistant for almost all the clinical PIs.

### 4.3. Mutations that alter the dimer interface

A distinct category of resistance mutations alters the dimer interface and stability. Residues Leu24, Ile50 and Phe53 lie at the interface between the two subunits. Leu24 does not interact directly with substrate or inhibitor, although this residue is adjacent to the catalytic Asp25. Ile50 and Phe53 are located in the flap. L24I is observed as a minor mutation in resistance to saquinavir, indinavir, nelfinavir, lopinavir and atazanavir. I50V is a major resistance mutation for fosamprenavir and darunavir, and a minor mutation for lopinavir. F53L appears as a minor resistance mutation selected by lopinavir and atazanavir. Dimer stability studies based on sensitivity to urea showed that mutations L24I, I50V and F53L result in proteases that are only ∼50% stable *i.e.* half of the activity is lost at a urea concentration that is 50% lower than seen for the wild type protease [67,28,61]. Also, the dimer dissociation was increased for these variants, unlike for tested variants with other mutations. The crystal structures of these variants showed altered interactions at the dimer interface. Reduced subunit-subunit interactions appear in structures of variants L24I and I50V with indinavir. The side chain of L24I lies in an internal hydrophobic cluster and the mutant shows reduced intersubunit interactions with the side chain of Phe99, which is consistent with the increased dimer dissociation.

**Figure 6.**
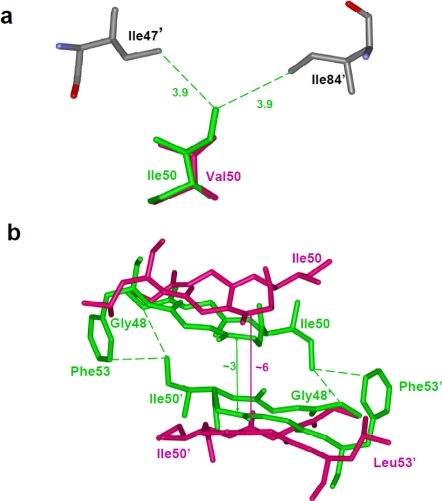
(a)The I50V mutation at the tip of the flap results in loss of intersubunit interactions with Ile47’ and Ile84’ [61]. Ile50 (PDB ID: 1SDT) and Val50 (PDB ID: 2AVS) are represented as green and magenta sticks, respectively. (b) The F53L variant eliminates intersubunit hydrophobic interactions between residues 53 and Ile50’ [28]. This loss of interaction is accompanied by a wider separation of the flaps. The wild type (PDB ID: 1HHP) and F53L flaps (PDB ID: 2G69) are shown in green and magenta sticks. The separation between the flaps is indicated in Å.

Ile50 sits at the tip of the flap and interacts with the second flap in the protease dimer and also with the inhibitor. In mutant I50V the changes at the dimer interface are accompanied by loss of interactions with inhibitor. The I50V variant has been studied with indinavir, darunavir and saquinavir [61,68,67]. Studies of the high resolution crystal structure with indinavir show that I50V mutation results in loss of intersubunit interactions (Figure 6a), which is consistent with the observed lower stability and higher dimer dissociation constant [61]. In addition, some of the direct van der Waals contacts with indinavir were abolished by the substitution further explaining the 50-fold weaker inhibition relative to that of wild type protease [61]. Structural studies of I50V mutant with darunavir exhibit a similar mechanism of resistance [57]. In addition to reduced hydrophobic interactions, the substitution results in loss of two hydrogen bonds between darunavir and the main chain of Asp30, in agreement with reduced susceptibility of this mutant to darunavir. The most severe loss of protease interactions with inhibitor is seen for darunavir and agrees with the selection of I50V in resistance to darunavir therapy and not with the other two PIs.

The variant with F53L was crystallized in the absence of inhibitor and the dimer structure shows the open conformation of the flaps. The side chain of Phe53 in the wild type protease dimer forms hydrophobic interactions with Ile50 from the other subunit. This interaction is eliminated in the F53L mutant leading to a wider separation of the two flaps (Figure 1 and 6b). The loss of interflap interactions agrees with the measurable (∼ 5nM) dimer stability of this mutant, which may partly contribute to resistance.

### 4.4. Distal mutations that transmit changes to the active site cavity

Mutations at the distal regions also affect the efficiency of PI drugs. Diverse and subtle structural changes have been observed for the protease variants with resistance mutations that alter residues outside of the active site cavity. This category includes mutation of flap residues and other residues without direct contacts with inhibitors or intersubunit contacts. Distal mutations are often observed together with other resistance mutations.

Flap mutant I54M is selected as a major drug resistant mutation in treatment with darunavir, although residue 54 has no direct interactions with inhibitors. The structure of the I54M variant has been analyzed with darunavir and saquinavir [67]. Mutation of residue 54 induces changes in residues 80–82 (the 80’s loop) that interact with inhibitors. In case of variant I54M, the 80’s loop is shifted away from residue 54 due to increased side chain length resulting in weaker interactions with darunavir. In contrast, the I54V variant has no significant change in interactions with darunavir or saquinavir. Alternatively, I54V, which is a minor resistance mutation for several PIs, shows an unusual structural change in the absence of inhibitor as described later.

**Figure 7.**
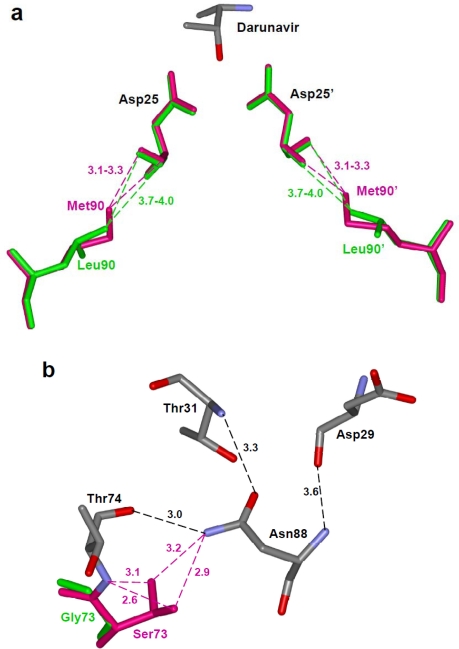
(a) The longer methionine side chain of Met90 in the L90M variant forms shorter van der Waals interactions with the main chain of catalytic Asp25, unlike the Leu90 in the wild type protease [68]. The wild type (PDB ID: 2IEN) and mutant protease (PDB ID: 2F81) are shown in green and magenta colored sticks, respectively. Part of darunavir is shown colored by element type. (b) The protease variant with G73S (PDB ID: 2AVV) substitution forms new hydrogen bond interactions of Ser73 with Thr74 and Asn88 (magenta dashed lines) [61]. The hydrogen bond network of Thr74, Asn88, Asp29 and Thr31 propagates the effects to the active site cavity.

Residue Leu90 is located in a hydrophobic pocket adjacent to the catalytic residues. L90M is a major resistance mutation for saquinavir and a minor mutation for almost all the other PIs. Structural studies of this mutant with different inhibitors reveal identical changes. The longer methionine side chain of L90M forms a shortened van der Waals contact, or C-H…O interaction, with the carbonyl oxygen of catalytic Asp25, which is not seen for Leu90 in the wild type protease (Figure 7a) [69,64,63]. Despite the absence of direct contact with the inhibitor, the new contacts with the catalytic loop alter the affinity for inhibitors. Molecular dynamics studies envisage that the perturbation of D25, T26 and G27 by the L90M mutation can cause additional conformational changes at the flap region and Pro79’ [70]. Also, the combination of a distal mutation with a mutation at the active site cavity can enhance the level of resistance to PI. The *in vitro* studies combining two major mutations D30N and L90M show that the protease variant maintains 40% of protease activity and exhibits high level of resistance to nelfinavir [63]. The crystal structure of the D30N/L90M double mutant shows a weaker hydrogen bond interaction of nelfinavir with the mutated Asn30 than with Asp30 in wild type protease [63].

Residue Gly73 is in a distal region far from the catalytic center of the protease, however, G73S is selected as a minor resistant mutation for atazanavir, fosamprenavir, indinavir, lopinavir and saquinavir. The variant with G73S shows altered specificity for substrate peptides, unlike many of the resistance mutations. The structure of G73S mutant in complex with indinavir identified a domino effect caused by this mutation that propagates to the active site [61]. The Ser side chain of residue 73 in the mutant forms new hydrogen bond interactions with Thr74 and Asn88. These residues, in turn, form hydrogen bond interactions with Asp29 and Thr31 closer to the active site cavity (Figure 7b). Since Asp29, Asp30 and Val32 interact directly with inhibitors and substrates, this intrasubunit network of hydrogen bonds induces resistance by correlated changes moving from the distal site to the active site [61]. The altered hydrogen bonding also leads to abnormal cleavage rates of peptides representing different polyprotein sites, which is likely to affect viral replication.

N88D in the distal region of protease is selected as major resistance mutation for atazanavir and a minor resistance mutation for nelfinavir. The crystal structure of the variant with D30N (major) and N88D (minor) double mutations in complex with nelfinavir, however, does not show significant structural change in the mutation sites when compared with wild type protease [63]. Analysis of patterns of mutations in 582 viruses with D30N mutations in 460 patients found N88D facilitates the co-occurrence of two major resistance mutations D30N and L90M in nelfinavir-failing patients and emphasizes the importance of the N88D substitution. Urea denaturation studies revealed that N88D mutation increases the stability of protease and compensates for the 2-fold decrease in stability due to another major resistance mutation L90M [60]. This increased stability results in better viral replication even in the presence of the inhibitor. In comparison to active site mutations that directly interact with the inhibitor fewer structural studies have focused on the distal mutations. Therefore, more unusual mechanisms for drug resistance may be discovered in future.

### 4.5. Reduced interaction with intermediate

**Figure 8.**
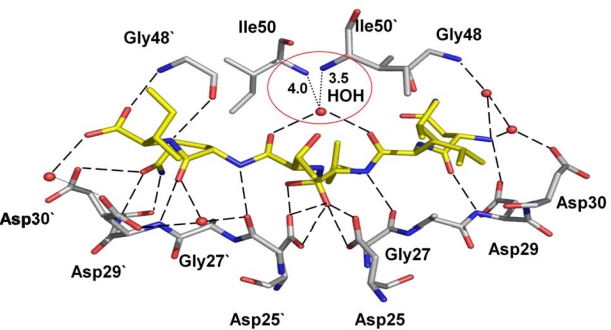
The variant I54V (PDB ID: 2B80) with peptide tetrahedral intermediate has lost the water mediated interactions with Ile50 and Ile50’, as indicated by red circle, in comparison to the wild type protease interactions in Figure 2 [32]. The hydrogen bond interactions between the protease and the intermediate are shown as broken lines.

Mutations of flap residue Ile54, including I54V, are attributed to resistance against all PIs, except for nelfinavir. Ile54 does not interact directly with inhibitor. Instead, the crystal structure of variant I54V in the absence of inhibitor shows an unusual molecular mechanism for resistance. The mutant has lost hydrogen bond interactions with the tetrahedral reaction intermediate of a peptide in the crystal structure [32]. Specifically, the water mediated hydrogen bonds linking the peptide intermediate and the flap residues Ile50 and Ile50” are lost in this mutant structure (Figure 8 and 2). The mutant structures with inhibitors show another effect. The structure of I54V darunavir complex reveals a shift in the 80’s loop, although no significant overall change occurs in the interactions between protease and the inhibitor [67]. These studies indicate that weaker binding of the inhibitor due to the I54V substitution may contribute to drug resistance.

## 5. Studies with clinical isolates containing multiple mutations

Clinical isolates with resistance to PIs typically contain multiple mutations in the protease. Structural interpretation of clinical isolates harboring multiple mutations presents the combined contributions from all individual mutations. Synergic effects due to different molecular mechanisms described in the previous sections produce proteases that are highly resistant to current clinical drugs. Structural data are described for several examples. For example, a deca-mutant protease containing K20R, V32I, L33F, M36I, I54V, L63P, A71V, V82A, I84V and L90M substitutions exhibits 1000 fold reduced susceptibility compared to wild type enzyme for indinavir [71]. The deca-mutant contains both active site and non active site mutations. Similarly, kinetic studies on a clinical isolate with 18 mutations L10I, I13V, K14R, V32I, L33F, K43T, M46I, I47V, I54L, I62V, L63P, A71T, I72T, G73T, V77I, P79S, I84V, L90M show 2000 and 400-fold weaker inhibition by darunavir and amprenavir, respectively [72]. The crystal structure of another clinical isolate with 19 mutations including T12V, I13V, I15V, K20M, V32I, L33F, K43T, I54L, K55N, I62V, L63P, A71V, I72V, G73S, V77I, V82L, I84V, L89V, L90M in complex with darunavir reveals the loss of two hydrogen bonds between darunavir and the protease backbone. Also, a shift in the aminophenyl moiety attributed to the coexistence of V32I and I84V induces loss of a water mediated interaction [72]. This mutant has 2000-fold weaker inhibition by darunavir when compared with wild type protease in agreement with the structural data. In another study, the crystal structure of a multi drug resistant variant with 10 mutations exhibits an expanded active site with the flaps separated by 8 Å more than seen in the wild type enzyme [73,74]. However, this mutant protease also includes the D25N inactivating catalytic mutation, which may influence the unusual separation of the flaps and expanded active site. Investigation of a pediatric clinical isolate with substitutions K20R, V32I, L33F, M36I, L63P, A71V, V82A, L90M, in addition to the engineered flap M46I, I54V and active site I84V mutations, shows a large decrease in binding affinity for nelfinavir [71]. The mutations resulted in conformational change in the 80’s loop, although the flap region remains largely unchanged. Since the structural changes in a clinical variant with large number of mutations are due to the combined effect of all the substitutions, it is difficult to identify the contribution of any single mutation. The effect of mutations in the active site cavity like V32I, V82A and I84V can be readily seen, but contributions of distal compensating mutations are difficult to infer. The effects of non-active site mutations are better investigated in protease variants with a single substitution, without the noisy background of multiple resistance mutations. Thus, the observed structural differences should be attributed to the entire set of studied mutations in the isolate.

## 6. Structure-guided design of PIs for resistant HIV protease

Analysis of the structures and activities of drug resistant variants of HIV-1 protease has revealed distinctive molecular mechanisms of drug resistance. These studies form a solid foundation for continued success in structure guided design of antiviral PIs targeting resistant strains of HIV. Several design strategies are derived from analysis of protease structures. The earlier PIs, which were designed to target wild type HIV-1 protease, retain a peptide-like chemical backbone. One major objective in the continued design of antiviral inhibitors is reduction of the peptidic character. Pursuing this goal, later PIs amprenavir, tipranavir and darunavir include sulphonamide replacing the peptide-like carbonyl group [38,39]. One proposed strategy is to design inhibitors that fit within the envelope formed by bound substrates since protease cleavage of its substrates is essential for viral replication. This strategy results in tight-binding (subnanomolar) inhibitors of HIV protease variants [75–77]. Targeting the flaps in the open conformation of the unliganded protease dimer is a unique approach with some successes for high affinity inhibitors. Novel chemical scaffolds are employed in two different studies. Pyrrolidine-based inhibitors bind between the open flaps in the protease crystal structure [78]. Unconventional inorganic metallacarboranes that bind the open flaps of the protease inhibit drug resistant variants [79].

**Figure 9.**
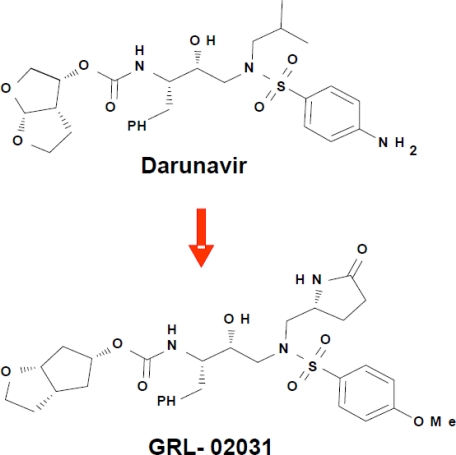
Chemical structures of darunavir and the new antiviral inhibitor GRL-02031.

The most successful strategy to date for targeting resistant HIV infections is exemplified by darunavir [58,5,6]. The critical feature of this strategy is incorporating new hydrogen bond donor or acceptor groups in the inhibitor to form hydrogen bond interactions with conserved regions of HIV protease. Interactions with the main chain amide and carbonyl oxygen are important since they cannot easily be altered by mutation. Similarly, specific amino acids are conserved and required for the protease structure or activity, and these also cannot be mutated without loss of activity. Therefore, targeting the main chain and conserved regions of the protease provides tight binding inhibitors that are less susceptible to the development of resistance. Pursuit of this strategy led to the development of darunavir, which was approved for AIDS salvage therapy in 2006 (Figure 9) [39,30,80].

Darunavir has proved exceptionally effective against drug resistant strains of HIV-1. Compared to earlier PIs, darunavir has a high genetic barrier to resistance and is active against resistant HIV isolates making it a valuable treatment option for treatment experienced or naive patients [81–83]. In addition to the usual binding of antiviral inhibitors in the active site of HIV protease, a second distinct binding site on the flexible flap was discovered for darunavir, which is potentially a novel target for the design of antiviral agents [68,84]. Darunavir binds to wild type HIV-1 protease using strong hydrogen bond interactions with conserved amino acid side chains and main chain atoms [39,30]. Analysis of crystal structures has demonstrated that darunavir retains the majority of these strong interactions when bound to resistant variants of HIV-1 protease [30,57,68,67]. Furthermore, darunavir and two new antiviral inhibitors, GRL-06579A and GRL-89065, have almost identical interactions with HIV-2 protease, consistent with their antiviral potency on both HIV-1 and HIV-2 [85]. A series of diverse antiviral inhibitors use the successful strategy of optimizing the hydrogen bond interactions with conserved regions of the HIV protease structure, as verified by crystal structures of inhibitor complexes with HIV-1 protease. These antiviral inhibitors display a variety of substituents at the P2, P2’ and P1’ groups on the darunavir-like sulfonamide scaffold and show nM inhibition or better [86–89,6]. In one example, the novel inhibitor GRL-02031 with modified P2, P1’ and P2’ groups (Figure 9) has similar antiviral potency to indinavir and saquinavir and, unlike those drugs, retained its potency on multidrug resistant HIV [6].

## 7. Conclusions

The development of antiviral inhibitors of HIV protease is one of the great success stories of structure based drug design. This strategy has resulted in several PIs that have increased the treatment options and improved viral suppression. However, the emergence of drug resistance and increasing numbers of drug resistant infections has affected the long-term potency of clinical drugs. Sustained efforts are necessary to discover the next generation of drugs that can combat drug resistant infections. Analysis of a number of drug resistant mutant structures in complex with clinical drugs has identified several molecular mechanisms by which the virus evades PIs. Generally, specific mutations show similar effects in complexes with several PIs including loss of protease interactions with inhibitor and/or loss of intersubunit interactions altering dimer stability. Furthermore, the structures of protease variants crystallized without inhibitor show some unique features contributing to drug resistance. As with the earlier generation of drugs these structural details are essential in the design of newer inhibitors to overcome drug resistance. The latest drug, darunavir, was designed to form hydrogen bonds with the backbone of the protease to ensure greater resilience to resistance as there is minimal change in the backbone conformation between wild type and mutant proteases. Further development of novel potent PIs incorporating this design concept and lessons learned from elucidating the mechanisms of drug resistance through structural studies is essential in the future treatment of AIDS.

## Figures and Tables

**Table 1.** Resistance mutations of HIV-1 protease. Residues in the substrate binding site are in blue. Resistance mutations for which structural information is available are in red.

Drugs	Major mutations						Minor mutations																																					
							L	V	I	G	K	L	D	V	L	E	E	M	K	M	I	G	I	F	I	Q	D	I	L	I	H	A	G	T	L	V	V	N	I	I	N	L	L	I
							10	11	13	16	20	24	30	32	33	34	35	36	43	46	47	48	50	53	54	58	60	62	63	64	69	71	73	74	76	77	82	83	84	85	88	89	90	93
	G	L					L					L													I			I				A	G			V	V		I					
Saquinavir/r	48	90					10					24													54			62				71	73			77	82		84					
	V	M					I					I													V			V				V	S			I	A		V					
							R																		L							T					F							
							V																														T							
																																					S							
	M	V	I				L				K	L		V				M							I							A	G		L	V							L	
Indinavir/r	46	82	84				10				20	24		32				36							54							71	73		76	77							90	
	I	A	V				I				M	I		I				I							V							V	S		V	I							M	
	L	F					R				R																					T	A											
		T					V																																					
	D	L					L											M		M												A				V	V		I		N			
Nelfinavir/r	30	90					10											36		46												71				77	82		84		88			
	N	M					F											I		I												V				I	A		V		D			
							I													L												T					F				S			
																																					T							
																																					S							
	I	I					L							V						M	I				I								G		L		V						L	
Fosamprenavir/r	50	84					10							32						46	47				54								73		76		82						90	
	V	V					F							I						I	V				L								S		V		A						M	
							I													L					V												F							
							R																		M												T							
							V																														S							
	V	I	V				L				K	L			L					M	I		I	F	I					L		A	G		L				I				L	
Lopinavir/r	32	47	82				10				20	24			33					46	47		50	53	54					63		71	73		76				84				90	
	I	V	A				F				M	I			F					I	V		V	L	V					P		V	S		V				V				M	
		A	F				I				R									L	A				L							T												
			T				R																		A																			
			S				V																		M																			
																									T																			
																									S																			
	I	I	N				L			G	K	L		V	L	E		M		M		G		F	I		D	I		I		A	G				V		I	I	N		L	I
Atazanavir/r	50	84	88				10			16	20	24		32	33	34		36		46		48		53	54		60	62		64		71	73				82		84	85	88		90	93
	L	V	S				F			E	M	I		I	I	Q		I		I		V		L	L		E	V		L		V	C				A		V	V	S		M	L
							I				R				F			L		L				Y	V					M		I	S				F							M
							V								V			V							M					V		T	T				T							
							C																		T							L	A				S							
																									A																			
	L	I	Q	T	V	I	L		I		K						E	M	K	M					I						H							N					L	
Tipranavir/r	33	47	58	74	82	84	10		13		20						35	36	43	46					54						69							83					90	
	F	V	E	P	L	V	V		V		M						G	I	T	L					A						K							D					M	
					T						R														M																			
																									V																			
	I	I	L	I				V						V	L						I														T							L	L	
Darunavir/r	50	54	76	84				11						32	33						47														74							89	90	
	V	M	V	V				I						I	F						V														P							V	M	
		L																																										

**Table 2.** Ki values for the inhibition of PR mutants by selected clinical inhibitors. Inhibition values were measured using different substrates and assay conditions and may not be directly comparable to each other.

Mutant	Inhibitor	*K*_i_ (nM)	*K*_i_/*K*_i_(WT)	Reference
L24I	Indinavir	1.4	2.6	[61]
D30N	Darunavir	6.6	30.0	[68]
D30N	Indinavir	7.0	32.0	[68]
G48V	Darunavir	17.0	29.0	[67]
G48V	Saquinavir	36.0	86.0	[67]
I50V	Darunavir	18.0	82.0	[68]
I50V	Saquinavir	36.0	164.0	[68]
I50V	Indinavir	27.0	50.0	[61]
I54V	Darunavir	5.0	8.6	[67]
I54V	Saquinavir	6.0	14.3	[67]
I54M	Darunavir	1.6	2.8	[67]
I54M	Saquinavir	2.2	5.2	[67]
F53L	Indinavir	11.1	20.6	[28]
G73S	Indinavir	0.55	1.0	[61]
V82A	Darunavir	1.3	1.3	[30]
V82A	Saquinavir	4.3	1.1	[59]
V82A	Indinavir	1.8	3.4	[64]
I84V	Darunavir	3.2	3.2	[30]
I84V	Saquinavir	4.3	1.1	[59]
D30N/N88D	Nelfinavir	7.0	100.0	[63]
N88D	Nelfinavir	0.2	2.9	[63]
L90M	Darunavir	0.03	0.14	[68]
L90M	Indinavir	0.09	0.17	[64]
